# NM23-H1 Expression of Head and Neck Squamous Cell Carcinoma in Association With the Response to Irradiation

**DOI:** 10.3389/fonc.2021.646167

**Published:** 2021-03-30

**Authors:** Yi-Fen Wang, Yi-Ke Lin, Chin-Ping Lin, Yu-Jen Chen, Chun-Ju Chang

**Affiliations:** ^1^ Department of Otorhinolaryngology and Head and Neck Surgery, Taipei Veterans General Hospital, Taipei, Taiwan; ^2^ Department of Medicine, National Yang Ming Chiao Tung University, Taipei, Taiwan; ^3^ Department of Food Science, National Taiwan Ocean University, Keelung, Taiwan; ^4^ Department of Medical Research, MacKay Memorial Hospital, Taipei, Taiwan; ^5^ Institute of Traditional Medicine, National Yang Ming University, Taipei, Taiwan; ^6^ Department of Radiation Oncology, MacKay Memorial Hospital, Taipei, Taiwan; ^7^ Department of Nursing, MacKay Junior College of Medicine, Nursing and Management, Taipei, Taiwan; ^8^ Department of Medical Research, China Medical University Hospital, Taichung, Taiwan

**Keywords:** Head and neck squamous cell carcinoma, NM23-H1, metastasis, radiation, clinical outcome

## Abstract

A low NM23-H1 expression in head and neck squamous cell carcinoma (HNSCC) was found to be associated with poor clinical outcome. Therefore, we investigated the role of NM23-H1 in the susceptibility of HNSCC cells to irradiation and its clinical significance. An *in vitro* study was also conducted to validate the results. Furthermore, we used immunohistochemistry to analyze NM23-H1 expression found in specimens of 50 HNSCC patients with cervical metastases receiving postoperative radiotherapy. Low tumor NM23-H1 expression was associated with locoregional recurrence of HNSCC (p=0.040; Hazard ratio=5.62) and poor clinical outcome (p=0.001; Hazard ratio=4.90). To confirm the effect of NM23-H1 on radiation-induced cytotoxicity, we generated several stable clones derived from a human HNSCC cell line (SAS) using knockdown and overexpression of NM23-H1. Knockdown of NM23-H1 decreased the radio-sensitivity of SAS cells, possibly associated with a decrease in the radiation-induced G2/M-phase accumulation and upregulation of cyclin B1. On the contrary, overexpression of NM23-H1 can reverse the aforementioned adverse results. Consequently, we suggest that NM23-H1 expression may be considered as a potential therapeutic treatment option for HNSCC patients.

## Introduction

The prevalence of Head and neck cancers (HNC), mainly squamous cell carcinomas (HNSCC), are increasing worldwide (1). HNSCC patients’ prognosis hasn’t been able to improve due to early metastases and poor response to chemoradiotherapy. Most HNSCC patients with potentially removable tumors also have lymphatic metastases at the time of their diagnosis (2). At present, there are no useful biomarkers for treatment planning for HNSCC patients with metastases.

Low NM23-H1 expression is long known to be associated with therapeutic resistance and metastasis in some cancers (3–6). However, clinical observation on NM23-H1 expression in treatment outcome of various cancers had yielded inconsistent results (7–10). Metastatic cancer cells were considered as unstable cells originated from the primary tumor, which could derive from a low-NM23-H1-expression (11, 12). NM23-H1 expression may decrease slow overtime during the course of tumor development (13). The survival mechanism of spreading cells was the major contributor to therapeutic resistance of recurrent tumors (4). Therefore, restoring NM23-H1 expression might lead to better treatment outcome of residual cancer cells (13).

There were very few studies on the clinical significance of NM23-H1 expression in cancer patients with metastasis receiving postoperative radiation. In our previous study, we discovered a low NM23-H1 expression of HNSCCs was associated with lymphatic metastases. The metastatic colonies were noted to have a reduced protein level compared to their neighboring normal cells (11). To investigate the effect of NM23-H1 expression on remaining tumor receiving adjuvant therapy, we analyzed the correlation between NM23-H1 expression and clinical pathological factors in HNSCC patients with cervical metastases treated with postoperative radiotherapy. To verify the effect of NM23-H1 on irradiation-induced cytotoxicity in HNSCC cells, we generated stable clones derived from a human HNSCC SAS cell line by knockdown and overexpression of NM23-H1 (14). The aim of the present study was to evaluate the impact of NM23-H1 on radio-sensitivity of HNSCC cells.

## Patients, Materials, and Methods

### Patients and Surgical Specimens

The surgical specimens were collected from fifty HNSCC patients and their cervical metastases were treated by surgery and postoperative irradiation between 1984 and 1998. Patient’s age ranged between 30-88 years with a median of 50 years. Written informed consent was obtained from all patients and this study was approved by the Institutional Review Broad of Taipei Veterans General Hospital. The present workup and treatment include general physical examination, computed tomography (CT) scan of head and neck, intraoral soft tissue biopsy, chest radiography, abdominal sonography, and whole-body radioisotope bone scan. Same treatment plan was given to all patients including postoperative local irradiation. Cancer staging was defined by the multidisciplinary head and neck cancer tumor board based on the sixth edition of American Joint Commission on Cancer TNM system. During the surgery, tumor tissues and the normal tissues (the neighboring grossly disease-free mucosa of surgical margins) were collected and examined by the surgical pathologist. Postoperative radiotherapy was given to patients due to the presence of insecure or positive resection margins, multiple metastatic lymph nodes, extracapsular spread, and perineural invasion. Furthermore, tumor recurrence was confirmed by clinical examinations. The median follow-up period was 65.7 months with a range of 3-218 months. For final analysis, 24 out of 50 patients survived and were considered free of HNSCC. The cumulative survival rates at 1-, 3- and 5-year were 76%, 54%, and 51%, respectively.

### Immunohistochemistry and Scoring

Expression of NM23-H1 in the pathologic tissues was observed and evaluated as previously described (2, 14).

### Irradiation on SAS Cells

In the previous study, we generated a few stable clones derived from a human HNSCC SAS cell line by using knockdown and overexpression of NM23-H1 (14). SAS_shRNAnm23_ (carrying nm23-H1 shRNA) and SAS_shRNA_ (carrying the pSuper plasmid) clones were obtained. Stable SAS clones expressing ectopically introduced HA-tagged NM23-H1 and harboring a control plasmid were also established and designated as SAS_nm23_ and SAS_control_. By using the Western blot, NM23-H1 protein level found in the mock controls (SAS_shRNA_ and SAS_control_) were not different from the parental SAS cells, whereas NM23-H1 protein level of SAS_shRNAnm23_ decreased by about 75% compared to the mock control (SAS_shRNA_). Overexpression of the ectopically introduced HA-tagged NM23-H1 was noted (14).

SAS cells were plated in 6-cm dishes at a density of 2.0 × 10^5^ cells/dish for 24 hours and cells were exposed to irradiation at different doses in a single fraction (sham RT, 1, 2 and 4 Gy). Six MeV of electron beam energy was delivered by a linear accelerator (Clinac^®^ 1800, Varian Associates, Inc., CA; dose rate 2.4 Gy/min). For each fraction, full electron equilibrium was reached by using a parallel plate PR-60C ionization chamber (Capintel, Inc., Ramsey, NJ).

### Colony Formation Assay of SAS Clones for Radiation Survival

Viable SAS cells were plated and allowed to grow in McCoy’s 5A medium containing 20% heat-inactivated FCS and 0.24% agarose at 37°C. After incubation of 10 to 14 days, the dishes were stained with 0.4% ≥ 50 cells were counted. The surviving fraction was presented as mean colonies/(cells inoculated × plating efficiency). The efficiency of control plating for SAS cells was approximately 60%. Survival curves were plotted using a linear-quadratic model. The sensitizer enhancement ratio (SER) was computed by using the required irradiation dose divided by the radiation dose needed for NM23-H1 over expression plus the irradiation dose needed to yield a surviving fraction of 37%.

### Western Blot Analysis and Cell Cycle Analysis

After exposure to radiation, cells were analyzed according to methods reported previously (11).

### Statistical Analysis

To examine the associations between NM23-H1 expression and each clinical- pathologic parameter, Chi-square (χ2) tests with Yates correction or Fisher’s exact test were performed. For prognostic analyses, Kaplan−Meier method was used to plot survival curves. Log-rank test was applied to examine the significant difference in survival between the patient groups. The collective effects of clinical-pathological factors were further analyzed by Cox proportional hazards model.

For *in vitro* studies, data were presented as the mean ± standard error in the three independent experiments. Differences between groups at each specific time frame were identified by one-way analysis of variance (ANOVA) or Wilcoxon-signed rank test. Statistical comparison between two independent variables was determined by two-way ANOVA followed by Dunnet’s test. This study used Statistical Package of Social Sciences (SPSS) software (SPSS Inc., Chicago, IL) for all statistical analyses. Probability P-values < 0.05 were considered statistically significant.

## Results

### Low NM23-H1 Expression in HNSCC Tumors Was Associated With Poor Prognosis of Patients Treated With Postoperative Radiation

In order to understand the role of NM23-H1 in prognosis of HNSCC patients with cervical metastases treated by surgery and postoperative irradiation, we inspected the NM23-H1 expression found in the specimens. By immunochemistry, NM23-H1 proteins were mostly localized in the cytoplasm while some were found in the nucleus (**Supplementary Material**). We focused mainly on nuclear expression of NM23-H1 in consideration of recent findings (15, 16). The interpretation of NM23-H1 expression was performed by two investigators (Wang YF and Chang CJ) unaware of the clinical data and kappa statistics revealed excellent agreement (kappa=0.79; p<0.001). In the discrepant cases, a final opinion was made based on two investigators’ consensus. The clinical significance of tumor NM23-H1 expression was assessed in comparison with clinical-pathologic features including age, primary tumor size, nodal involvement of neck, distant metastasis, and tumor recurrence (**Table 1**). The analysis showed more patients (17/31) with NM23-H1-negative tumors had locoregional recurrence compared to those (4/19) with NM23-H1-positive tumors (p=0.040). Eighty-six percent (12/14) patients with distant metastasis had NM23-H1-negative tumors while 53% (19/36) patients without distant metastasis had NM23-H1-negative tumors. Overall, patients with distant metastasis appeared to have a higher rate of NM23-H1-negative tumors compared to those without distant metastasis with a marginal significance of p=0.067.

**Table 1:** Relationship between NM23-H1 expression in head and neck squamous cell carcinoma and clinicopathologic parameters of 50 patients with resectable cervical metastasis treated by postoperative radiation.

Clinicopathologic parameters	Number of patients	Interpretation of tumor NM23-H1 expression		
		Negative (31)	Positive (19)	p value^1^
**Age (years)**				
≤50	28	18	10	0.935
>50	22	13	9	
**Primary tumor size**				
≤4 cm	31	22	9	0.171
>4 cm	19	9	10	
**Metastatic lymph node(s)**				
=1	10	4	6	0.216
>1	40	27	13	
**Distant metastasis**				
Negative	36	19	17	0.067
Positive	14	12	2	
**Tumor recurrence**				
Negative	29	14	15	0.040
Positive	21	17	4	

^1^Based on Chi-square test with Yates′ (continuity) correction.

**Figure 1:**
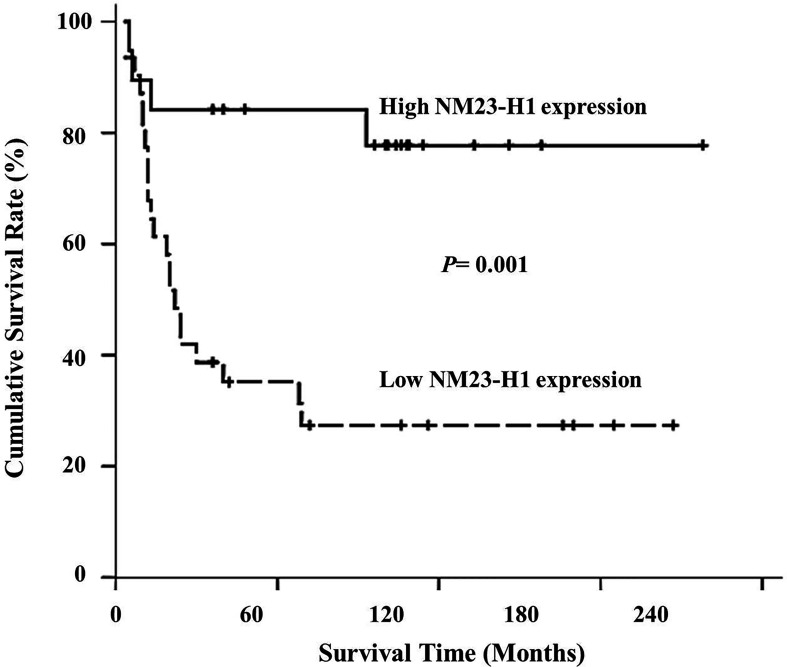
Overall survival curve of 50 patients with head and neck squamous cell carcinoma treated by postoperative radiation associated with NM23-H1 expression of primary tumors. Patients with NM23-H1-positive tumors had significantly longer survival time than those with NM23-H1-negative tumors (p < 0.01).

To confirm whether a low NM23-H1 level affects treatment outcome, we evaluated the prognostic relevance of NM23-H1 expression in HNSCC patients. When conducting univariate analyses using log-rank tests, patients with recurrence (p<0.001), distant metastasis (p<0.001) and negative NM23-H1 expression in primary tumors (p=0.001) were shown to have poorer survival. By immunohistochemistry, patients with NM23-H1-negative tumors appeared to have a less desirable outcome than those with NM23-H1-positive tumors (**Figure 1**). During multivariate analyses using a Cox proportional hazard model, distant metastasis (p=0.011) and tumor recurrence (p=0.012) remained as independent factors associated with patients’ prognosis. However, tumor NM23-H1 expression was found not significantly correlated with patients’ prognosis (**Table 2**).

**Table 2:** Survival analysis of 50 patients with head and neck squamous cell carcinoma with resectable cervical metastasis treated by postoperative radiation.

Clinicopathologic parameters (Number of patients analyzed)	p value	
	Univariate analysis^1^	Multivariate analysis^2^
**Age (years)**	0.236	
≤50 (28) >50 (22)		
**Primary tumor size**	0.086	
≤4 cm (31) >4 cm (19)		
**Metastatic lymph node(s)**	0.181	
=1 (10) >1 (40)		
**Distant metastasis**	< 0.001	0.011
Negative (36) Positive (14)		
**Tumor recurrence**	< 0.001	0.012
Negative (29) Positive (21)		
**NM23-H1 expression in primary tumor**	0.001	0.171
Negative (31) Positive (19)		

^1^Based on Log-rank test.

^2^Based on Cox proportional hazards model.

### Knockdown of NM23-H1 Attenuated the Susceptibility of SAS Cells to Radiation

**Figure 2:**
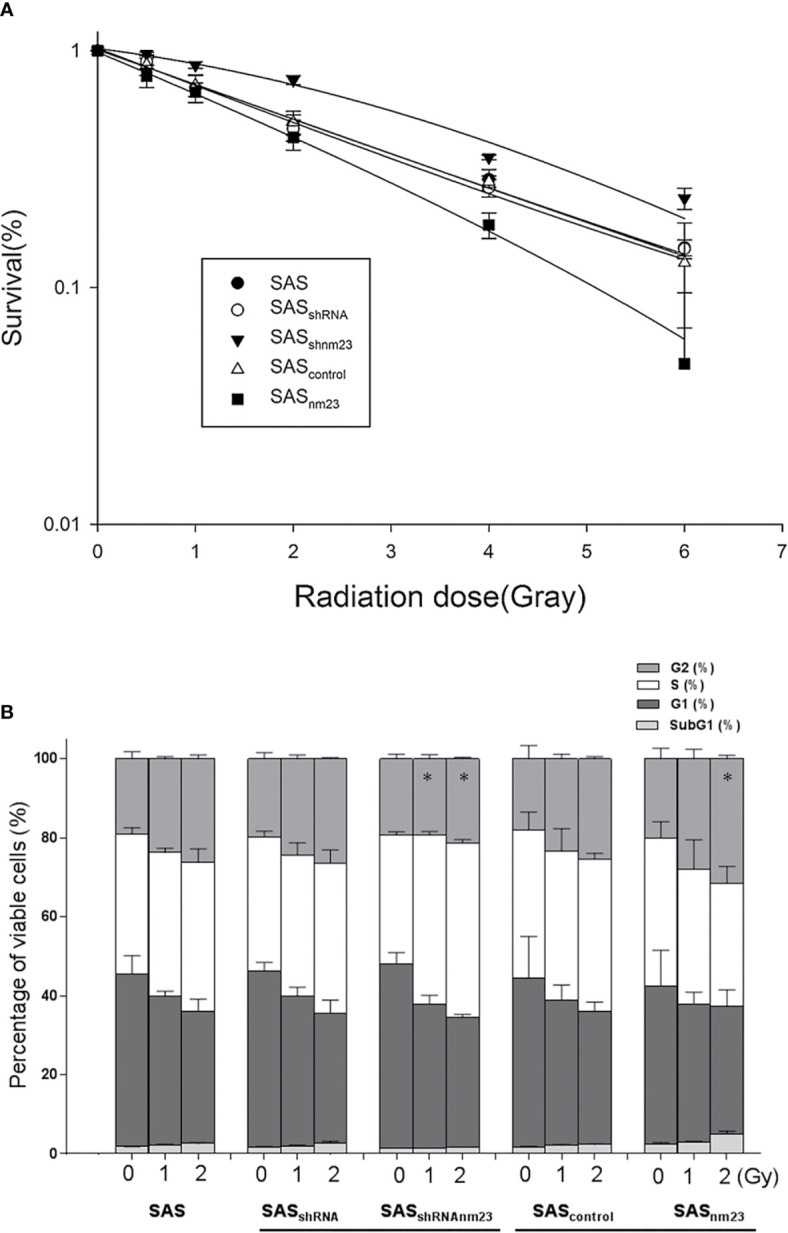
Knockdown of NM23-H1 attenuated the susceptibility of SAS cells to irradiation and decreased the G2/M-phase cell accumulation. **(A)** Cell viability and radiation survival curves. **(B)** Cell cycle analysis. Data represent the mean ± standard error of three independent experiments. *p < 0.05 compared with mock controls; statistical significance was determined using the paired t-test.

To clarify the role of NM23-H1 in radiosensitivity of SAS cells, cell viability was examined using colonogenic assays following irradiation. The survival fraction of NM23-H1-knockdown (SAS_shRNAnm23_) cells seemed higher than that of the mock control (SAS_shRNA_) at 2 Gy, 4 Gy and 6 Gy, indicating that knockdown of NM23-H1 attenuated radiosensitivity of SAS cells. Conversely, the survival fraction of NM23-overexpressing (SAS_nm23_) cells was significantly lower than that of the mock control (SAS_control_) when they were treated with radiation doses at 2 Gy, 4 Gy and 6 Gy (**Figure 2A**). Overexpression of NM23-H1 slightly enhanced the radiation response of SAS cells with a maximal sensitizer enhancement ratio (SER) of 1.3, whereas knockdown of NM23-H1 attenuated the radiosensitivity with a SER of 0.7.

### Neither Knockdown Nor Overexpression of NM23-H1 Significantly Affects Radiation-Induced Apoptosis

To confirm the impact of NM23-H1 expression on radiation-induced apoptosis of SAS cells, we assessed the percentage of apoptotic cells by DNA fragmentation, TUNEL assays and annexin V-propidium iodide (PI) staining. Among SAS clones with different levels of NM23-H1 expression, no significant difference was found in the amount of apoptosis after irradiation (data not shown). Our findings suggested that classic apoptosis may not be the primary pathway of radiosensitization associated with NM23-H1 expression in SAS cells (17, 18).

### Knockdown of NM23-H1 Decreased the Proportion of Radiation-Induced SAS Cell Accumulation at the G2/M Phase

SAS cells were treated with graded radiation doses (0, 1, and 2 Gy) and the cell cycle was analyzed by flow cytometry. Exposure to radiation caused an increase in the percentage of SAS cells at G2/M phase of the cell cycle (19). Knockdown of NM23-H1 diminished the proportion of SAS_shRNAnm23_ cells in radiation-induced G2/M-phase arrest compared to the mock control (SAS_shRNA_). In contrast, overexpression of NM23-H1 enhanced post-irradiation G2/M-phase accumulation of SAS_nm23_ cells compared with the mock control (SAS_control_) (**Figure 2B**).

### Knockdown of NM23-H1 Downregulated Cyclin E and A and Upregulated Cyclin B1 and D1

**Figure 3:**
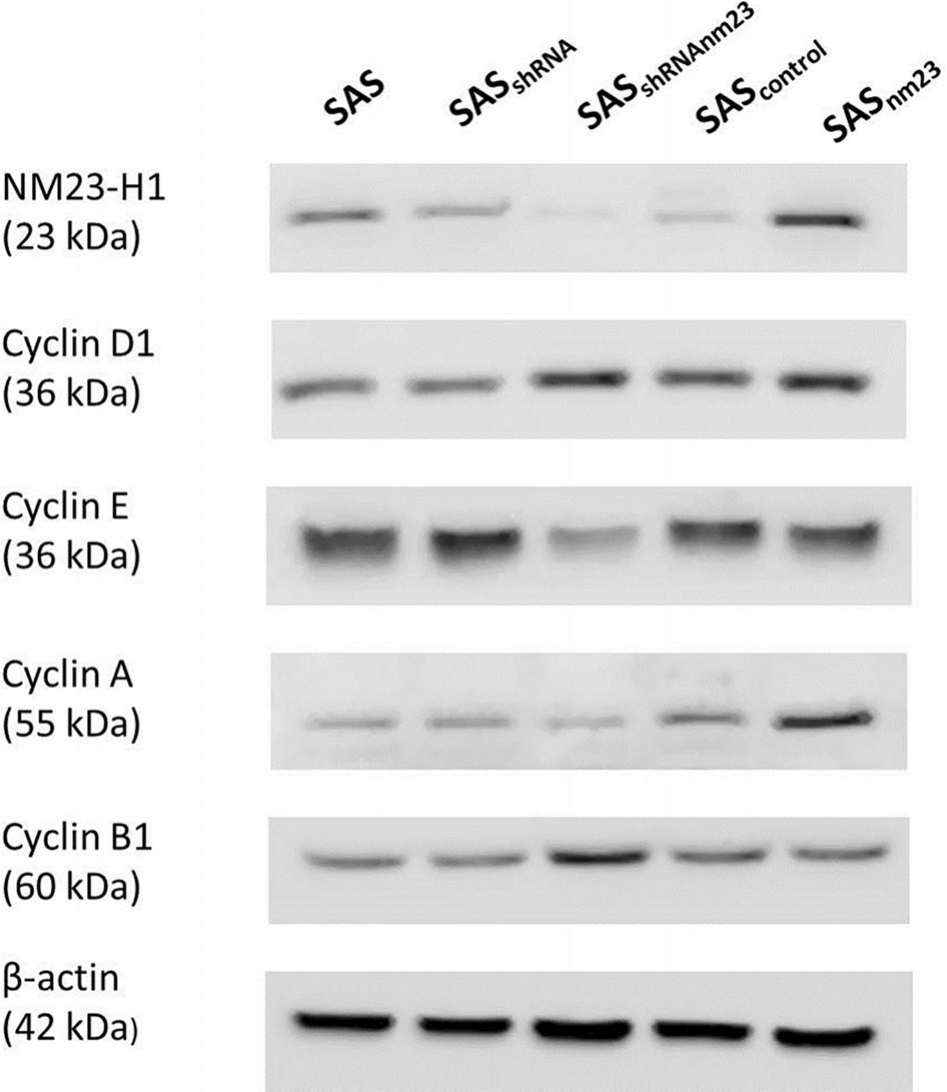
Western blot of NM23-H1 and cyclin D1, E, A1, and B1 in the SAS head and neck squamous cell carcinoma clones. Knockdown of NM23-H1 downregulated cyclin E and A, and upregulated cyclin D1 and B1 in SAS_shRNAnm23_ cells, compared with SAS_shRNA_. SAS, parent SAS clone; SAS_shRNA_, mock knockdown clone; SAS_shRNAnm23_,NM23-H1 knockdown clone; SAS_control_, mock overexpression clone; SAS_nm23_, NM23-H1 overexpression clone.

To convey the physiologic relevance of NM23-H1 proteins in SAS cells, we examined whether NM23-H1 is involved in modulating the expression of cyclin D1, E, A and B1. Prior to irradiation, knockdown of NM23-H1 downregulated cyclin E and cyclin A and slightly increased cyclin B1 and cyclin D1, compared to the mock controls. These findings supported the fact that NM23-H1 may involve in modulating cell cycle (**Figure 3**).

**Figure 4:**
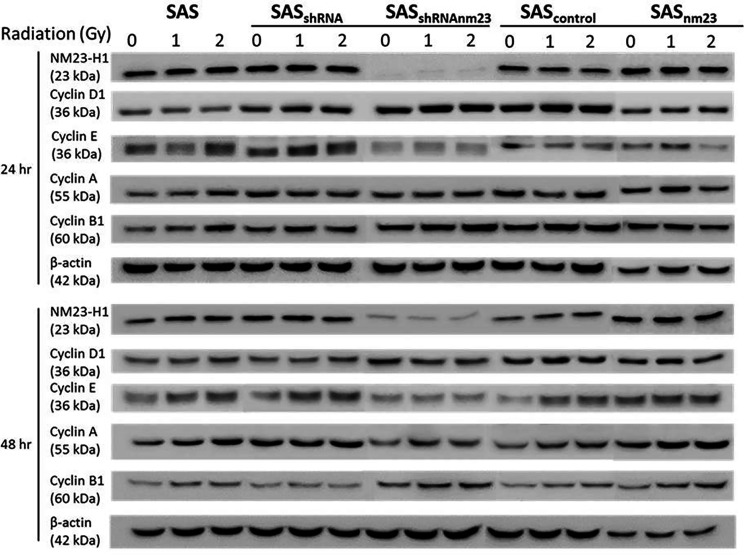
Western blot showing the effect of NM23-H1 expression on cyclin D1, E, A1, and B1 after SAS cells treated by irradiation. Following irradiation at graded doses of 0, 1 and 2 Gy, cells were collected, lysed, and analyzed by Western blot for SAS clones, including parental (SAS), mock knockdown (SAS_shRNA_), NM23-H1 knockdown (SAS_shRNAnm23_), mock overexpression (SAS_control_), and NM23-H1 overexpression (SAS_nm23_) cells.

Following the exposure to irradiation, SAS cells displayed a slight increase in cyclin B1 levels compared to the controls without irradiation. However, we did not observe other significant effects of NM23-H1 expression on the protein levels of cyclin D1, E and A in response of SAS cells to irradiation (**Figure 4**).

## Discussion

The salient findings of this study showed low NM23-H1 expression in primary tumors was associated with locoregional recurrence in HNSCC patients with cervical metastases receiving surgery and radiotherapy. Cells with low NM23-H1 expression were less susceptive to irradiation compared to those with high NM23-H1 expression. We also found that irradiation can induce DNA damage and G2/M-phase arrest in SAS cells, eventually resulting in cell death. Knockdown of NM23-H1 reduced the radiosensitivity of SAS cells through diminished irradiation-induced G2/M-phase arrest, possibly due to the upregulation of cyclin B1.

We recently reported a low NM23-H1 expression could decrease cisplatin sensitivity of HNSCC cells (14). However, many HNSCC patients with cervical metastases only underwent post-operative radiation without cisplatin-based chemotherapy. To investigate whether NM23-H1 expression has an impact on radiosensitivity, we evaluated the relationship between NM23-H1 expression and the response to irradiation in HNSCC patients with cervical metastases. Clinical data demonstrated a low tumor NM23-H1 expression was associated with poor survival and locoregional recurrence of HNSCC in patients with cervical metastases receiving postoperative radiation. Our finding is consistent with a previous report on patients with laryngeal squamous cell carcinoma (20). To the best our knowledge, there were limited researches discussing the role of NM23-H1 in response to postoperative irradiation in HNSCC patients with cervical metastases (14, 20). Our study found a correlation between low NM23-H1 expression and locoregional recurrence of HNSCC. The underlying mechanism could be attributed to patients’ poor response to irradiation.

Both clinical data and *in vitro* studies supported our hypothesis that NM23-H1 should be considered as one of the important factors in evaluating the susceptibility of HNSCC cells to radiotherapy. Our findings were in agreement with other previous reports showing a higher NM23-H1 expression in tumor had a favorable response to radiotherapy in laryngeal and nasopharyngeal cancers (21, 22). However, some publications reported NM23-H1 expression of cancer cells did not have an influence on cell growth kinetics, but the knockdown of NM23-H1 can induce therapy resistance by promoting the survival mechanism (23). We postulated that the influence of NM23-H1 on radiation-induced cytotoxicity may be related to DNA damage of the cells or the regulation of cell cycle in our tested cell line.

Radiotherapy achieves its therapeutic effects by prompting apoptosis and non-apoptotic cell death (24). In our study on SAS cells, no early apoptotic DNA fragmentation was observed after irradiation, suggesting typical apoptosis may not be the primary pathway for radiation-induced death. Nevertheless, we are unable to eliminate the possibility that NM23-H1 could have a role in the caspase-independent apoptotic pathway, where DNA is damaged by single-strand nicks under certain circumstances (25). Furthermore, we noticed most SAS cells exhibited growth arrest following irradiation, and this finding was similar to previous studies conducted on other cancers (26–28).

The cells at the late S-phase seem relatively radioresistant and those found at the G2/M-phase appear to be sensitive to radiation (29). Without radiation exposure, there was no significant difference in cell cycle distribution among the SAS clones established by knockdown and overexpression of NM23-H1 (6). Therefore, we proposed that the effect of NM23-H1 expression on tumor growth was triggered by the radiation-induced DNA damage in SAS cells. Most anticancer treatments exert their cytotoxicity through the cell cycle arrest and cell death is usually the outcome. Irradiation could cause SAS cells to be accumulated at the G2/M-phase, and this phenomenon is similar to how SAS cells with normal p53 function regulating the cell cycle (30). It is documented from other studies that NM23-H1 positively regulates p53 activities, and thus NM23-H1 may involve in radiation-induced cell cycle arrest (31, 32). Knockdown of NM23-H1 usually resulted in less SAS cells arresting at the G2/M-phase after irradiation. It is possible that some NM23-H1-knockdown cells may recover from irradiation-induced damage and return to the cell cycle. During fractionated radiotherapy, the aforementioned mechanism may prevent survived cells from becoming radiosensitive to the next radiotherapy fraction.

Certain reports indicated NM23-H1 might modulate the cell cycle regulators (33, 34). Prior to irradiation, knockdown of NM23-H1 in SAS cells upregulated cyclin B1 and cyclin D1 compared with the mock controls. Some studies mentioned an elevated expression of cyclin B1 or D1 conferred radioresistance, while reduced expression enhanced radiosensitivity (35, 36). In NM23-H1-knockdown cells, increased cyclin B1 protein presumably facilitates cell cycle progression, resulting in better survival than the mock controls (37). It was evident that NM23-H1 inhibited the activity of STAT3 *via* a negative feedback, and the inhibition of STAT3 downregulated cyclin D1, resulting in subsequent antitumor effects (38, 39). However, we did not observe any significant effects of NM23-H1 expression on cyclin B1 and D1 in SAS cells after irradiation (**Figure 4**). We suggest the NM23-H1 effect on radiation-induced G2/M arrest may not be associated with the post-radiation cyclins expression. Additional studies are warranted to clarify the link between NM23-H1 and radiation-induced G2/M arrest and radiocytotoxicity.

Our study found that knockdown of NM23-H1 downregulated cyclin A in SAS cells and this was consistent with a decreased cyclin A level noted in the transgenic NM23-M-knockout hepatoma (4). NM23-H1-knockdown SAS cells with a low cyclin A level exhibited less susceptibility to radiation compared with the mock control. However, we didn’t observed any significant effect of NM23-H1 on cyclin A after exposure to radiation. In SAS cells without irradiation, knockdown of NM23-H1 significantly downregulated cyclin E. High cyclin E expression was associated with cell cycle arrest at the G0/G1-phase, influencing cells’ response to radiotherapy (40). However, downregulation of cyclin E in NM23-H1-knockdown cells did not significantly affect cell cycle distribution at the G1-phase. Therefore, it is reasonable to assume that cyclin E may not be involved in NM23-H1-mediated radiosensitivity in SAS cells.

NM23 proteins acted as a scaffold in signal transduction (41). Loss of NM23 may cause genomic instability, contributing to the progression of cancer stem cells (40, 42). Upregulation of NM23 was found in tumor of mice under cotreatment of Paclitaxel and electro-acupuncture (43). Our study results may be a cell line-specific scenario. Further work is needed to thoroughly investigate the functional role of NM23-H1 in DNA damage. Furthermore, our results should be validated in other human HNSCC cell lines.

We followed the REMARK guidelines (44) and checked all items as far as possible based on our available data. One limitation of this study is retrospective design, so some details are difficult to be presented according to REMARK recommendations. Therefore, more investigation is needed for further validation. However, we believed such concerns do not interfere with our results for the prognostic significance of NM23-H1 in HNSCC patients.

In summary, clinical data demonstrated low NM23-H1 expression of cancer cells was associated with locoregional recurrence and poor prognosis of HNSCC patients after postoperative radiation. In vitro study, knockdown of NM23-H1 expression lessened radiation-induced cytotoxicity, whereas overexpression of NM23-H1 enhanced radiosensitivity. Knockdown of NM23-H1 upregulated cyclin B1 and cyclin D1 in SAS cells compared with the mock control. Following the exposure to radiation, knockdown of NM23-H1 decreased G2/M-phase cell accumulation compared with the mock control. Overexpression of NM23-H1 increased post-irradiation G2/M-phase cell cycle arrest compared with the mock control. Our study suggested that downregulated NM23-H1 expression may reduce radiosensitivity through decreased radiation-induced G2/M-phase arrest. Further research is warranted to clarify the link between NM23-H1 and other cell cycle regulators in the response of HNSCC cells to irradiation. As for clinical relevance, enhancing tumor NM23-H1 expression may potentially be a therapeutic strategy to improve the effectiveness of postoperative radiotherapy for HNSCC patients with cervical metastases.

## Data Availability Statement

The original contributions presented in the study are included in the article/**Supplementary Material**. Further inquiries can be directed to the corresponding author.

## Ethics Statement

The studies involving human participants were reviewed and approved by the Institutional Review Broad of Taipei Veterans General Hospital. The patients/participants provided their written informed consent to participate in this study.

## Author Contributions

Y-FW designed the research, analyzed data, and wrote this manuscript. Y-KL contributed in performing the research. C-PL contributed in performing the research. Y-JC revised this manuscript critically for important intellectual content. C-JC contributed in the design of the research, data analysis, and interpretation. All authors contributed to the article and approved the submitted version.

## Funding

All phases of this study were funded by the Ministry of Science and Technology of the Republic of China, grant number NSC102-2314-B-075-045-MY2; and the Taipei Veterans General Hospital (VGH Taipei), grant number V103C-147, V104C-073, V105C-137, and V106C-115.

## Conflict of Interest

The authors declare that the research was conducted in the absence of any commercial or financial relationships that could be construed as a potential conflict of interest.
