# Supplementary material for: NM23-H1 Expression of Head and Neck Squamous Cell Carcinoma in Association With the Response to Irradiation
Source: Front Oncol. 2021 Mar 30;11:646167. doi: 10.3389/fonc.2021.646167 (PMC8042278; doi:10.3389/fonc.2021.646167)
Supplement: Supplementary file 1 [file Image_1.pdf]

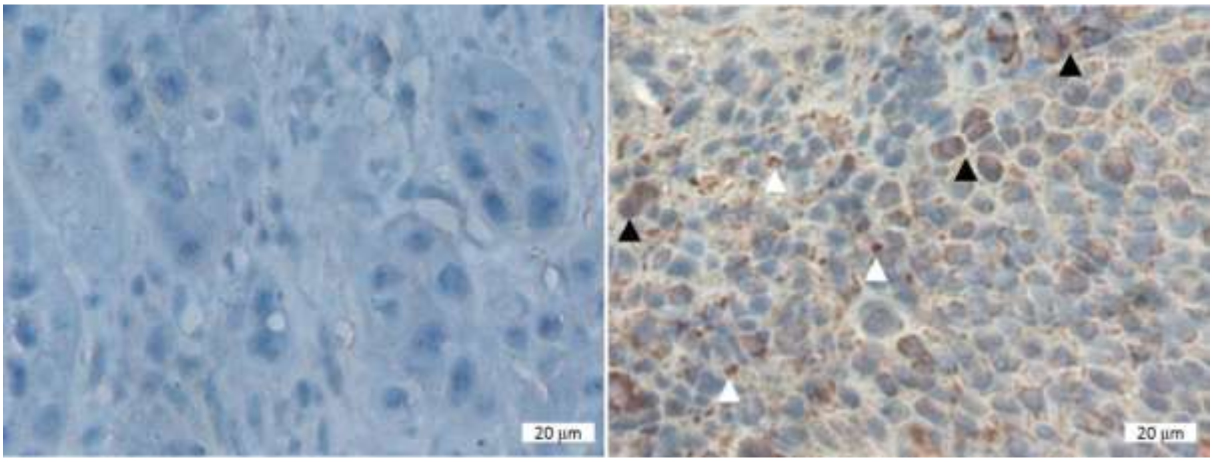

Supplementary material: **Immunohistochemical staining for NM23-H1 protein in the representative examples of head and neck squamous cell carcinoma (HNSCC).** NM23-H1 expression was interpreted as positive in the right panel, and negative in the left panel, respectively. In the right panel, nuclear staining marked with black arrows, and cytoplasmic staining marked with white arrows. The photographs were taken at the magnification of × 400.
